# Management change following NGS diagnosis of inborn errors of immunity: The Australasian experience

**DOI:** 10.70962/jhi.20260120

**Published:** 2026-10-01

**Authors:** Shruti Swamy, Bella Shadur, Hannah Hu, Kahn Preece, Kuang-Chih Hsiao, Annaliesse Blincoe, Jovanka King, Natasha Moseley, Andrew McLean-Tooke, Anna Sullivan, Peter McNaughton, Alberto Pinzon-Charry, Mariana Machado Melo, Alisa Kane, Lucinda J. Berglund, Dan Suan, Seth L. Masters, Ilia Voskoboinik, Satoshi Okada, Cindy S. Ma, Christopher C. Goodnow, Peter Hsu, Isabelle Bosi, Nicolás Urriola, Alex Stoyanov, Ming-Wei Lin, Jan Sinclair, Shannon Brothers, Theresa Cole, Joanne M. Smart, Sam Mehr, Stephanie Richards, Mark Taranto, Brynn Wainstein, Katie Frith, Paul E. Gray, Stuart G. Tangye

**Affiliations:** 1 Clinical Immunogenomics Research Consortium Australasia, Sydney, Australia; 2 Royal Prince Alfred Hospital, Camperdown, Australia; 3 Royal North Shore Hospital, St. Leonards, Australia; 4 Sydney Children’s Hospital, Randwick, Australia; 5 Faculty of Medicine and Health, University of Sydney, Camperdown, Australia; 6 Garvan Institute of Medical Research, Darlinghurst, Australia; 7 Faculty of Medicine and Health, University of New South Wales, Sydney, Australia; 8 The Royal Children’s Hospital, Parkville, Australia; 9 St. Vincents Hospital, Darlinghurst, Australia; 10 Campbelltown Hospital, Campbellton, Australia; 11 John Hunter Children’s Hospital, Newcastle, Australia; 12 Starship Child Health, Te Toka Tumai, Auckland, New Zealand; 13 Faculty of Medical and Health Sciences, University of Auckland, Auckland, New Zealand; 14 Women’s and Children’s Hospital Network, Adelaide, Australia; 15 Immunology Directorate, South Australia Pathology, Adelaide, Australia; 16 Adelaide University, Adelaide, Australia; 17 Perth Children’s Hospital, Perth, Australia; 18 Sir Charles Gairdner Hospital, Perth, Australia; 19 PathWest Laboratory Medicine, Perth, Australia; 20 Children’s Health Queensland, Brisbane, Australia; 21 The University of Queensland, Brisbane, Australia; 22 Griffith University, Nathan, Australia; 23 Institute of Molecular Biosciences, University of Queensland, St. Lucia, Australia; 24 Liverpool Hospital, Liverpool, Australia; 25 Westmead Hospital, Westmead, Australia; 26 NSW Health Pathology, Sydney, Australia; 27 Cellular Genomics Futures Institute, University of New South Wales, Sydney, Australia; 28 Centre for Innate Immunity and Infectious Diseases, Hudson Institute of Medical Research, Clayton, Australia; 29 Department of Molecular and Translational Science, Monash University, Clayton, Australia; 30 Killer Cell Biology Laboratory, Peter MacCallum Cancer Centre, Melbourne, Australia; 31 Sir Peter MacCallum Department of Oncology, University of Melbourne, Parkville, Australia; 32 Hiroshima University Graduate School of Biomedical and Health Sciences, Hiroshima, Japan; 33 The Children’s Hospital at Westmead, Westmead, Australia; 34 The Institute of Clinical Pathology and Medical Research, Westmead, Australia

## Abstract

Inborn errors of immunity (IEIs) are rare monogenic disorders with diverse, often life-limiting manifestations. Next-generation sequencing (NGS) has transformed IEI diagnostics, but data on therapeutic impact remain limited. We conducted a multicenter retrospective study through the Clinical Immunogenomics Research Consortium of Australasia, encompassing 12 hospitals across Australia and New Zealand. Probands with confirmed monogenic IEIs diagnosed by panel, whole-exome, or whole-genome sequencing were included. Among 205 probands with pathogenic variants in 86 genes, NGS findings prompted management changes in 84%: 71.7% major and 12.2% minor. Major changes included recommendation/planning for hematopoietic stem cell transplant (40.8%), initiation of targeted therapies (39.5%), and immunoglobulin replacement or prophylactic antimicrobials (18.4% each). Cascade testing identified 32 affected relatives, including four from probands who themselves did not have a discernible management change, demonstrating the broader impact of molecular diagnosis. Overall, NGS-based diagnosis drove substantial management changes and further defined the Australasian IEI landscape, supporting early genomic testing as a cornerstone of precision care.

## Teaser

Swamy et al. describe an Australian/New Zealand cohort where identification of monogenic inborn errors of immunity through next-generation sequencing resulted in management change in 84% of cases, including guiding hematopoietic stem cell transplants and initiating targeted treatment. Molecular diagnosis further enabled diagnoses of affected relatives, underscoring the value of early genomic testing in inborn errors of immunity.

## Introduction

Inborn errors of immunity (IEIs) are rare monogenic disorders with highly diverse clinical presentations, ranging from recurrent severe and potentially life-threatening infections to autoimmunity, autoinflammation, allergy, and malignancy that can affect a wide variety of organ systems (1, 2). Over 550 IEIs are now recognized (3), and access to next-generation sequencing (NGS) has transformed the landscape for early diagnosis of these disorders (4). The management of patients diagnosed with a monogenic IEI has profoundly improved in recent years, driven by advances not only in rates of genetic diagnoses, but also in understanding molecular mechanisms of disease pathogenesis (5), and the development and/or implementation of gene/pathway-guided targeted therapies (6, 7). This revolution has shifted care away from empiric management, which has been largely nonspecific and phenotype-driven, toward genetic etiology/mechanism-driven precision medicine that targets causal molecular pathways (6, 7).

Several international studies have assessed management change (MC) following definitive molecular diagnosis of a monogenic IEI in their patient cohorts, albeit with MCs being highly variable. For instance, an early study reported an MC in 25% of >100 patients with a variety of IEI phenotypes from 22 countries (8), compared with 67% in a South African study of patients with similarly mixed IEI phenotypes (9). Importantly, greater rates in MC have been reported in more recent studies, including 83% in a pediatric Italian center focused on autoimmune and allergic IEIs (10), 88% for an IPEX-like cohort (11), and 100% in an Australian pediatric center (12). Changes in management in these studies range from implementation of targeted pathway–specific therapies, to guiding curative treatment options such as hematopoietic stem cell transplant (HSCT), or surveillance and monitoring strategies based on known disease manifestations and progression of specific IEIs.

Access to genomic testing in Australia and New Zealand is complex and heterogeneous, ranging from publicly funded services to private immunogenetic options, depending on the state and location of the clinic. An Australia and New Zealand model for genetic healthcare delivery was established in 2015, encompassing a multiregion interdisciplinary collaborative network to provide diagnostic support to clinicians treating patients with IEIs. The Clinical Immunogenomics Research Consortium of Australasia (CIRCA) comprises clinicians and scientists bridging a variety of specialties and areas of expertise, including pediatric and adult clinical immunologists, molecular pathologists, genetic counselors, bioinformaticians, and laboratory scientists ([13], https://www.garvan.org.au/research/collaboration/circa). CIRCA currently comprises ∼250 members across 32 hospitals in Australia and New Zealand (combined population of 33.5 million people) that collectively cares for patients of all ages. CIRCA is also affiliated with nine research institutions, the peak professional body for clinical immunology and allergy in Australia and New Zealand (Australasian Society of Clinical Immunology and Allergy), as well as Australian and New Zealand patient support and advocacy organizations (Immune Deficiency Foundation Australia, Immune Deficiency Foundation of New Zealand). By engaging the collaboration within this geographically diverse consortium, we sought to capture the Oceanic experience on the therapeutic impact and rate of MC following delivery of a definitive molecular diagnosis in Australian and New Zealand patients with monogenic IEIs.

## Results

### Patient demographics and phenotypes

**Figure 1.**
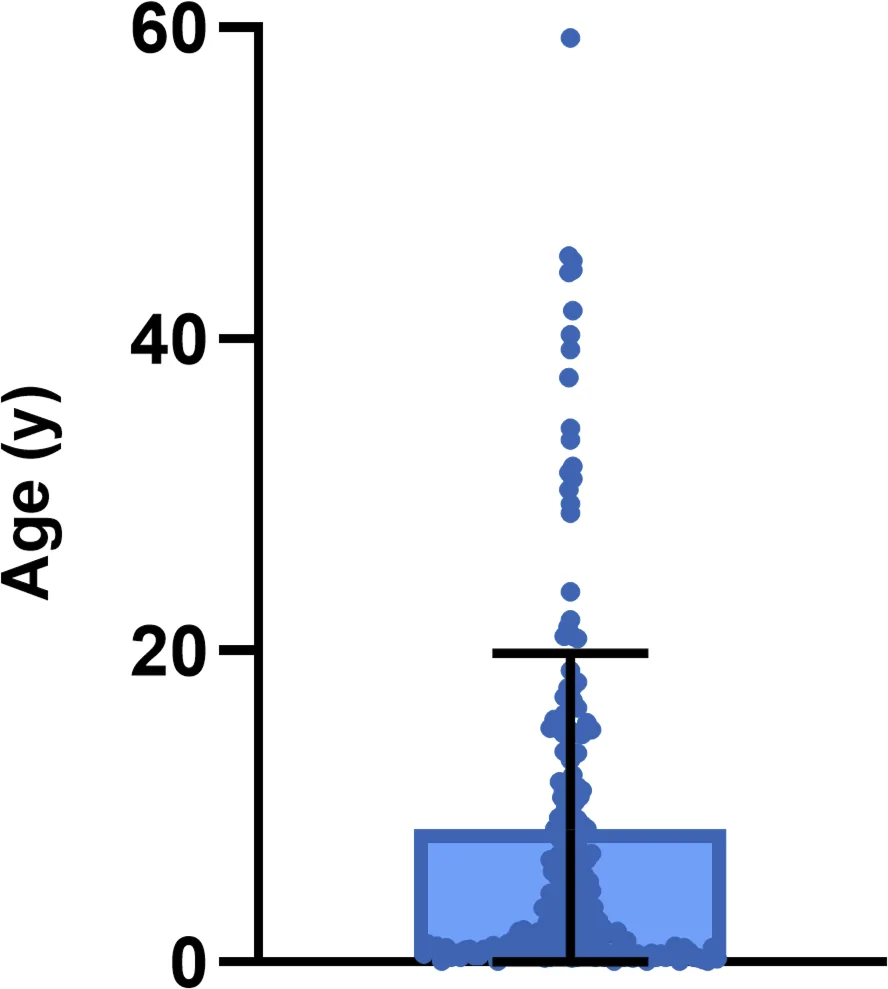
**Distribution of age at diagnosis.** Age of individuals at the time of genetic diagnosis. Box represents the mean; bars represent the standard deviation; dots represent ages of individual patients at diagnosis. y, years.

31 clinicians from 12 tertiary hospitals across five Australian states (New South Wales, Victoria, Queensland, Western Australia, and South Australia) and the North and South Islands of New Zealand contributed to this study. A total of 205 probands from 199 families were included, with pathogenic variants identified in 86 different genes, causing 88 different IEIs. The median age at genetic diagnosis was 3.75 years (range 0–59 years, Fig. 1), and 113 patients (55.1%) were male (Table 1). More than half of the patients (54.6%) were diagnosed under 5 years of age, with a further 34 (16.6%) diagnosed between 5 and 10 years of age, 34 (16.6%) between 10 and 20 years, 8 (3.9%) between 20 and 30 years, 9 (4.4%) between 30 and 40 years, and a further 8 (3.9%) diagnosed at over 40 years of age (Table 1). The earliest diagnosis via NGS in our cohort was in 2013, and the most frequent method of diagnosis was via an IEI gene panel (109 cases, 53.2%), followed by whole-exome sequencing (WES) (70 cases, 34.1%) and whole-genome sequencing (WGS) (26 cases, 12.7%). The most common immunological phenotype was immune dysregulation (60 cases, 30.8%), including atopy, hemophagocytic lymphohistiocytosis, hepatitis, primary sclerosing cholangitis, vasculitis, enteritis, colitis, cytopenias, lymphoproliferation, urticaria, autoimmune endocrinopathies, arthritis, periodic fevers, interstitial lung disease, encephalitis, transverse myelitis, uveitis, and alopecia. 58 cases (29.7%) presented only with recurrent and/or atypical infections, with pathogens including *Candida* spp., *Aspergillus* spp., *Pneumocystis jirovecii*, *Burkholderia cepacia*, *Pseudomonas* spp., persistent EBV, disseminated disease post-live attenuated viral vaccination, and nontuberculous mycobacteria. 49 cases (25.1%) presented with a combination of infections and immune dysregulation. 49 cases (25.1%) had extra-immune features with 17 (8.7% of the entire cohort; 34.7% of this *n* = 49 subgroup) presenting predominantly with extra-immune manifestations (including stroke, livedo reticularis, ataxia, short stature, intestinal atresia, skeletal dysplasia, pulmonary hypertension, pulmonary alveolar proteinosis, syndromic features, and cardiac, neurological, and renal abnormalities). 11 patients (5.6%) were identified through newborn screening (NBS) for severe combined immunodeficiency (SCID) (Table 1).

**Table 1.** Gender, age at diagnosis, method of molecular diagnosis, and clinical presentation of 205 IEI patients from Australia and New Zealand

​	Number (%)
Male	113 (55.1%)
Female	92 (44.9%)
**Age at diagnosis (years)**	​
<5	112 (54.6%)
5–<10	34 (16.6%)
10–<20	34 (16.6%)
20–<30	8 (3.9%)
30–<40	9 (4.4%)
>40	8 (3.9%)
**Method of diagnosis**	​
IEI gene panel	109 (53.2%)
WES	70 (34.1%)
WGS	26 (12.7%)
**Clinical presentation (*N* = 195)**	​
Immune dysregulation	60 (30.8%)
Infection	58 (29.7%)
Infection + immune dysregulation	49 (25.1%)
Predominantly extra-immune features	17 (8.7%)
NBS (SCID) test	11 (5.6%)

### Distribution of IEI phenotypes and causative genes

**Figure 2.**
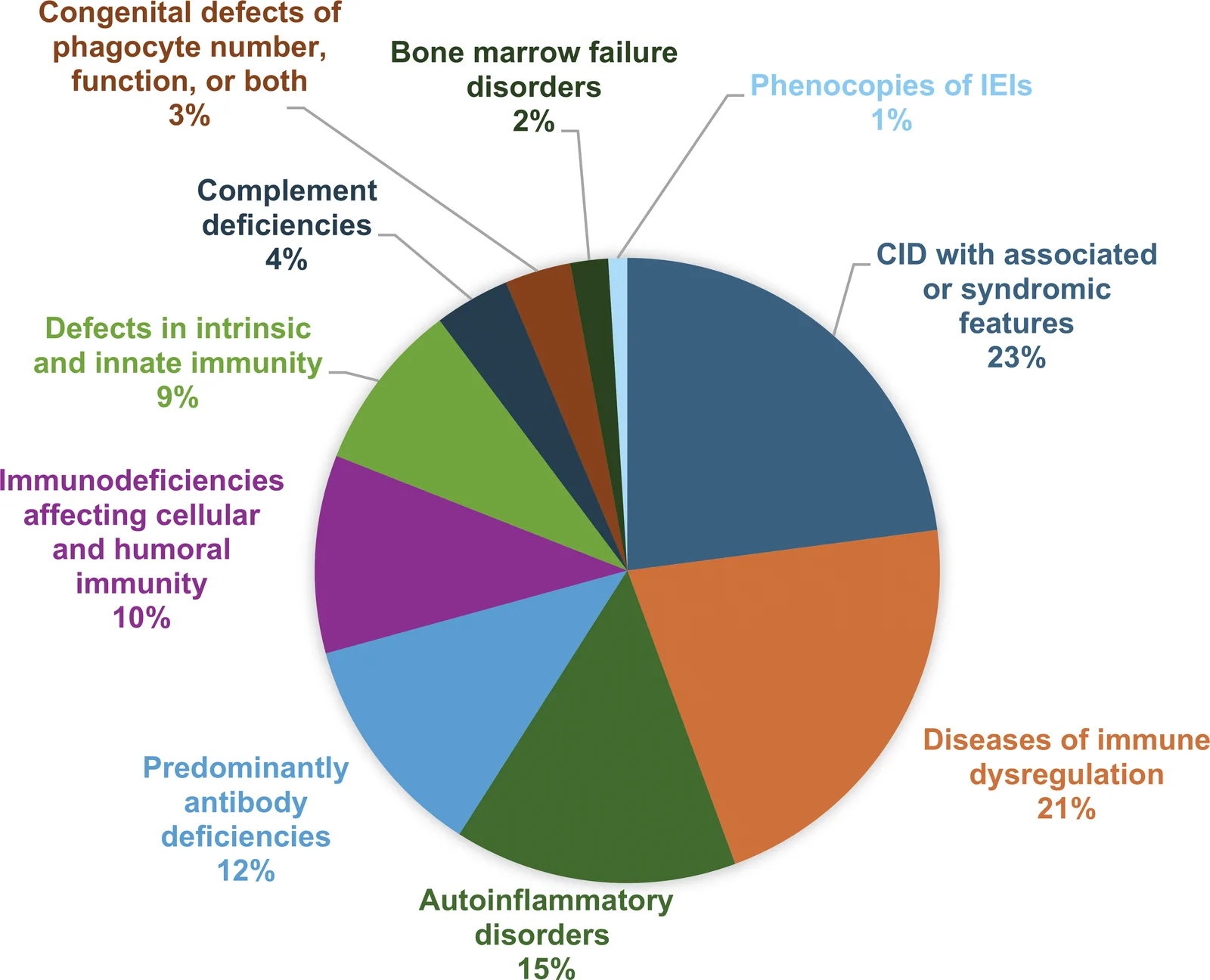
**Classification and frequency of genetic variants.** Genetic variants and affected patients were classified as per the IUIS phenotypic classification of human IEIs. Frequencies of affected genes were stratified into the 10 phenotypic classifications.

Using classification established by the International Union of Immunological Societies (IUIS) Expert Committee on IEIs (3), the most frequent IEIs were combined immunodeficiencies with associated or syndromic features (48 cases, 23.4%) and diseases of immune dysregulation (44 cases, 21.5%), followed by autoinflammatory disorders (27 cases, 13.2%), predominant antibody deficiencies (26 cases, 12.7%), immunodeficiencies affecting cellular and humoral immunity (21 cases, 10.2%), defects in intrinsic and innate immunity (18 cases, 8.7%), complement deficiencies (8 cases, 3.9%), congenital defects of phagocyte number or function (7 cases, 3.4%), bone marrow failure (4 cases, 2.0%), and phenocopies of IEIs associated with autoantibodies or somatic variants (2 cases, 1.0%) (Fig. 2).

**Figure 3.**
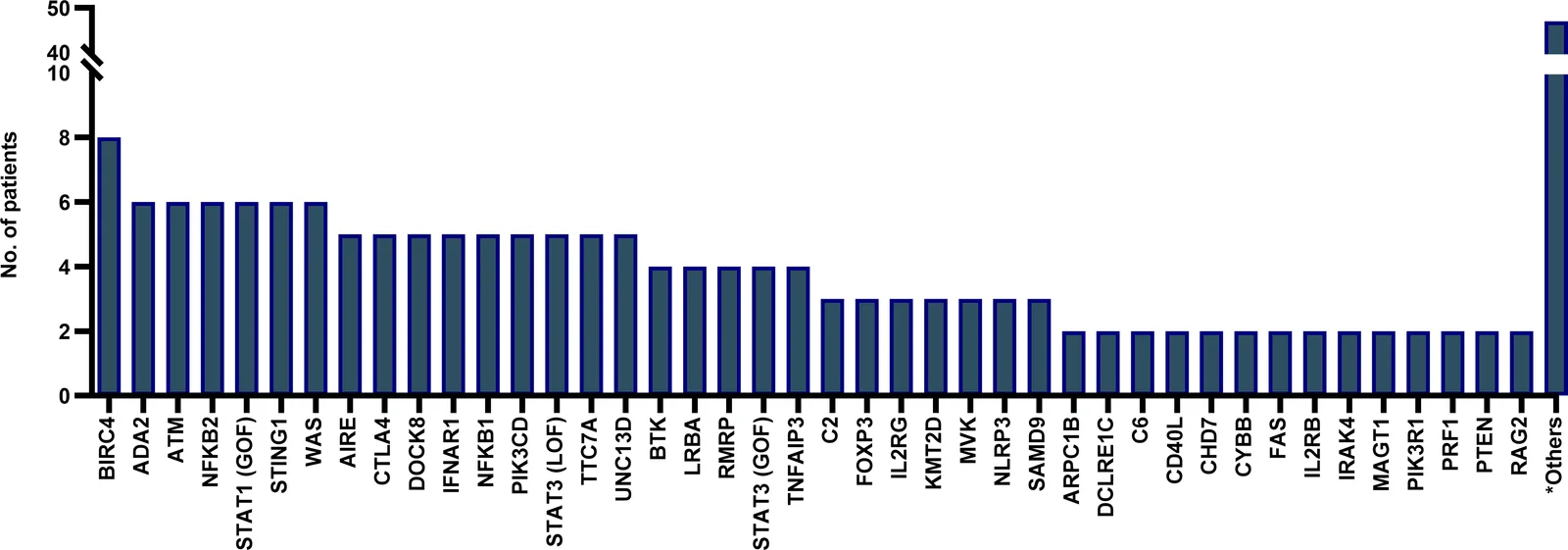
**Distribution and frequency of pathogenic variants in IEI patients.** Number of patients with variants in the indicated genes. *, genes with variants found in individual patients.

The most frequently reported pathogenic genotypes included *BIRC4* (encoding XIAP; 8 cases, 3.9%), followed by deleterious variants in *ADA2*, *ATM*, *NFKB2*, *STING1*, *WAS*, and *STAT1* (gain of function [GOF]), identified in 6 patients each (36 cases in total, 2.9% per gene, 17.6% of the total cohort); in *AIRE*, *CTLA4*, *DOCK8*, *IFNAR1*, *NFKB1*, *PIK3CD* (GOF), *STAT3* (dominant negative/loss of function [LOF]), *TTC7A*, and *UNC13D* in 5 patients each (45 cases in total, 2.4% per gene, 22.0% of the total cohort); *BTK*, *LRBA*, *RMRP*, *STAT3* (GOF), and *TNFAIP3* in 4 patients each (20 cases in total, 2.0% per gene, 10.0% of the total cohort); *C2*, *IL2RG*, *KMT2D*, *MVK*, *NLRP3*, *PIK3R1*, and *SAMD9* in 3 patients each (21 cases in total, 1.5% per gene, 10.2% of the total cohort); and finally *ARPC1B*, *C6*, *CD40L*,* CHD7*, *CYBB*, *DCLRE1C*, *FAS*, *FOXP3*, *IL2RB*, *IRAK4*, *MAGT1*, *PRF1*, *PTEN*, and *RAG2* in 2 patients each (28 cases in total, 1% per gene, 13.7% of the total cohort) (Fig. 3). An additional 46 genes were each represented by a single patient (22.4% of the cohort) (see Fig. 3 and Table S1).

### MC following confirmation of a genetic IEI diagnosis

**Figure 4.**
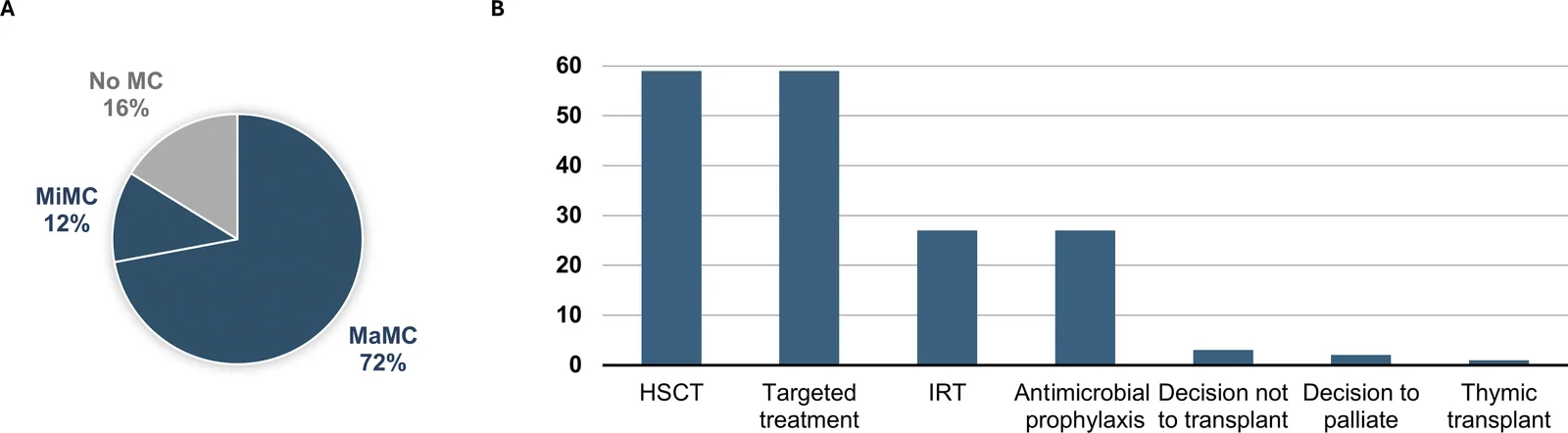
**MC resulting from a genetic diagnosis. (A)** Rates of MC (major: MaMC; minor: MiMC; no MC). **(B)** Numbers of individuals with specific MaMC.

**Figure 5.**
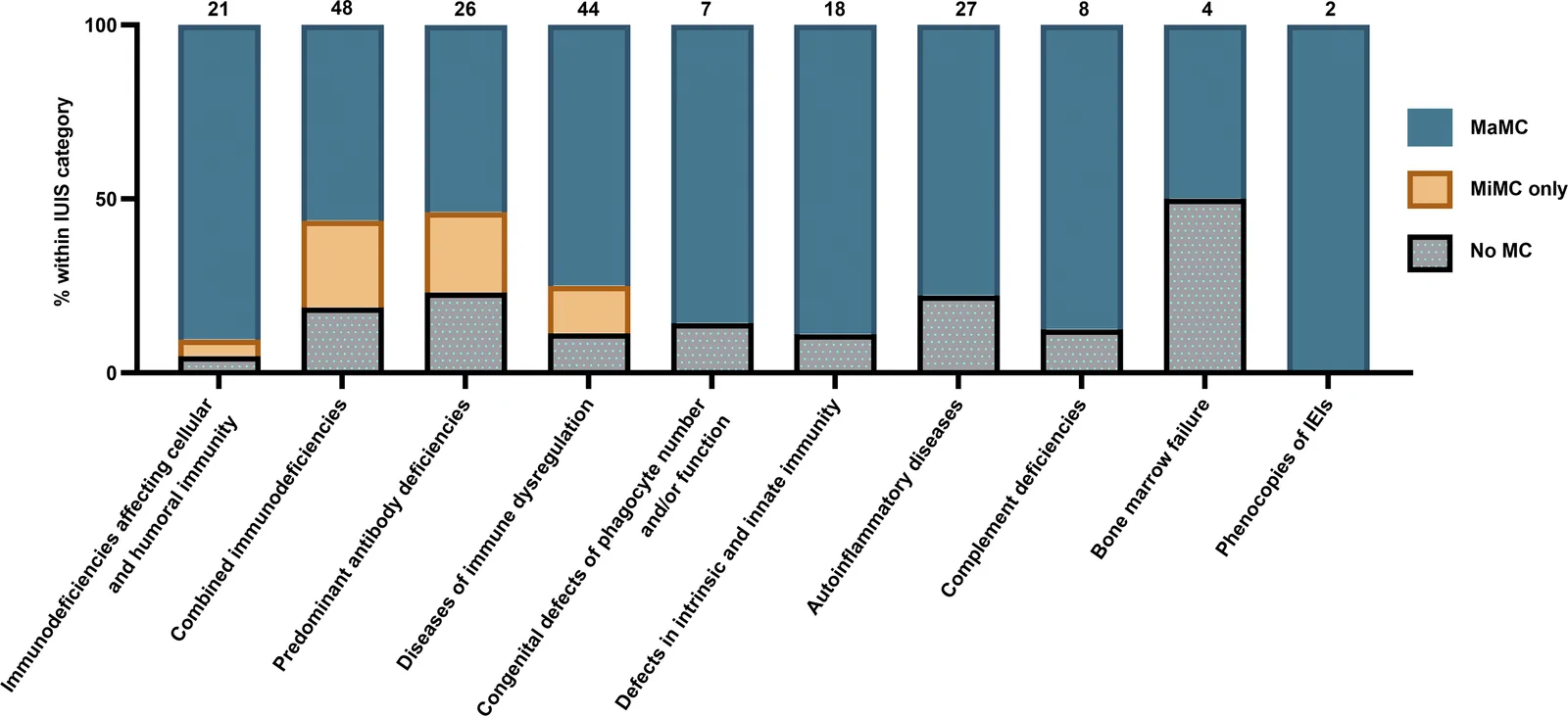
**Rates of MC within the 10 IUIS IEI categories.** Percentages of major (MaMC, blue), minor (MiMC, orange), and no MC (gray) resulting from a monogenic diagnosis within each of the 10 IUIS IEI categories. Numbers represent the total number of patients within each category.

MC occurring because of a definitive genetic diagnosis was seen in 172 (83.9%) individuals, with major management change (MaMC) in 71.7% (*n* = 147) and minor management change (MiMC) in 12.2% (*n* = 25) (Fig. 4 A). Importantly, a total of 32 relatives of the probands were diagnosed across the whole cohort. This included four family members of index cases where there was no individual MC despite a genetic diagnosis. This highlights that even in the absence of an MC, a molecular diagnosis remains highly informative and beneficial to the index patient(s) and their family. When patients were stratified across the 10 IUIS IEI categories (3), MC was observed in at least half of the individuals within each group, with the greatest proportion of MC seen in immunodeficiencies affecting cellular and humoral immunity (*n* = 20, 95.23% of the IUIS IEI category) and in phenocopies of IEIs (*n* = 2, 100% of the IUIS IEI category), followed by defects in intrinsic and innate immunity (*n* = 17, 88.9% of the IUIS IEI category), diseases of immune dysregulation (*n* = 39, 88.6% of the IUIS IEI category), complement deficiencies (*n* = 7, 87.5% of the IUIS IEI category), congenital defects of phagocytes (*n* = 6, 85.7% of the IUIS IEI category), combined immunodeficiencies (*n* = 39, 81.2% of the IUIS IEI category), autoinflammatory diseases (*n* = 21, 77.8% of the IUIS IEI category), predominant antibody deficiencies (*n* = 20, 76.9% of the IUIS IEI category), and bone marrow failure (*n* = 2, 50% of the IUIS IEI category) (Fig. 5). When stratified by age at diagnosis, MC was again observed across all age groups, and interestingly in at least 60% of patients within each age group (Table 2).

**Table 2.** Rates of MC, stratified by age at diagnosis

Age at diagnosis (years)	No. of patients with MC/total patients in age group	MC as a	
		% of all patients with MC (*n* = 172)	% within age group
<5	99/112	57.6%	88.4%
5–<10	26/34	15.1%	76.5%
10–<20	28/34	16.3%	82.4%
20–<30	6/8	3.5%	75%
30–<40	8/9	4.7%	88.9%
>40	5/8	2.9%	62.5%

The most common MaMC was recommendation and/or planning for HSCT (including surveillance, donor selection, and guiding conditioning regimens) and initiating/planning targeted treatment (*n* = 59 each, 40.1% of all MaMC). Others included initiating immunoglobulin replacement therapy (IRT; *n* = 27, 18.4%) and prophylactic antimicrobials (*n* = 27, 18.4%). There were also three cases where the diagnosis led to a decision not to progress to HSCT (*LRBA*, *BIRC4*, and somatic *KRAS*), two cases for whom the decision was made to withdraw active care and to palliate (both with biallelic *TTC7A* variants), and one patient who underwent thymic transplant (*CHD7*) (Fig. 4 B). Targeted treatments included abatacept (for *CTLA4*, *LRBA*), sirolimus (for *CTLA4*, *FAS*, *LRBA*, *NFKB1*, autosomal dominant (AD) *PIK3CD* GOF, AD *PIK3R1* LOF, *PTEN*, *STAT1* GOF), leniolisib (for AD *PIK3CD* GOF, AD *PIK3R1* LOF), anakinra (for *MVK*, *NLRP3*, *TNFRSF1A*), anifrolumab (for *STING1*), rituximab (for *AIRE*, *CXCR4* GOF, *MAGT1*,* UNC13D*), IFNγ treatment (for *IFNGR1*, *RORC*), TNF inhibitors (for *ADA2*, *OUTLIN*, *TNFAIP3*), JAK inhibitors (for *AIRE*, *OTULIN*, *STAT1* GOF, *STAT3* GOF, *STING1*, *TNFAIP3*, somatic *STAT5B* GOF*/KRAS* GOF), eculizumab (for *CFHR1–CFHR4*), darbepoetin (for erythropoietin, *HYOU1*), G-CSF (for *HYOU1*), enzyme replacement therapy (for *ADA*), ustekinumab (for *LAD1*), Creon (for *SBDS*,* SAMD9*), and steroids (for *MVK*,* OUTLIN*,* STING1*,* TNFAIP3*,* ADA2*).

All 11 infants who were identified as having a possible IEI due to a positive NBS test for SCID had an MC result based on their genetic diagnosis. This included tailored HSCT conditioning regimens (*n* = 7; *ARPC1B*, *DCLRE1C*, *IL2RG*, *IL7R*, *LIG1,RAG1*, and *RMRP*), and, as discussed above, thymic transplant (*n* = 1), decision to palliate (*n* = 2), and targeted treatment with G-CSF (*n* = 1).

## Discussion

With the rapid rise in identification of pathogenic variants and genes resulting in monogenic IEIs, as well as the expanding scope of targeted and pathway-directed treatment options, early access to genetic testing is critical to inform optimal management in selected patients. NGS allows for early identification of monogenic causes of IEIs, increasing the likelihood of meaningful MC in these patients. Our cohort of 205 patients with monogenic IEIs harbored variants in 86 distinct genes, highlighting the remarkable genetic diversity of IEIs and the need to consider a broad range of potential etiologies in this clinically heterogeneous population.

A MaMC prompted by molecular diagnosis in our cohort of IEI patients was the ability to direct and refine donor and conditioning selection for HSCT. Our data demonstrated that securing a molecular diagnosis by NGS led to substantial changes in clinical management in over 80% of cases, including >80% of infants identified as having a possible IEI by NBS for SCID. Several studies have demonstrated that obtaining a genetic diagnosis before HSCT in IEIs is generally associated with higher survival, fewer graft failures, and better event-free survival, largely enabled by earlier and better tailored HSCT (14, 15, 16). In the context of an abnormal NBS result for SCID, early monogenic diagnosis has been shown to refine risk stratification, delineate SCID from non-SCID patients, and enable targeted intervention (17, 18). Several international studies have demonstrated the feasibility and clinical utility of incorporating NGS into NBS as a concurrent first- or second-tier test, enabling earlier access to disease-specific interventions (19, 20, 21, 22). An Australian study further showed that WGS performed on newborn blood spots facilitated cascade testing and subsequent diagnoses in relatives and was associated with high parental acceptance and low decisional regret (23). Consequently, NGS is emerging as an important diagnostic tool for neonates with SCID NBS results suggestive of an IEI. In addition, a recent Australian study highlighted the potential of genomic-based NBS as a concurrent first-tier strategy to augment the diagnostic yield for identifying infants with IEIs (24).

Our study demonstrated that most patients within each IUIS IEI category had a MaMC resulting from their monogenic diagnosis, underscoring the broad impact of molecular testing across the full spectrum of IEIs. Isolated MiMC was relatively more frequent in patients with predominant antibody and combined immunodeficiencies, potentially reflecting the lower use of targeted therapies in these IEI subgroups with treatment largely guided by the clinical phenotype prior to monogenic diagnosis, such as hypogammaglobulinemia (IRT) and recurrent infections (prophylactic antibiotics). While a previous study reported MC resulting from a genetic IEI diagnosis in all IUIS categories apart from antibody deficiencies (8), we suggest that a change in screening and monitoring, classified as MiMC, contributes to improved long-term patient care and management of disease morbidity. Importantly, we also demonstrated that MC occurred in over 60% of individuals within all age groups following confirmation of a monogenic IEI. This adds to the growing body of evidence that beyond pediatric diagnoses, pursuing a monogenic diagnosis in appropriately selected adults can meaningfully inform clinical management, resolve diagnostic uncertainty, and provide valuable opportunities for prognostication and cascade testing (25, 26).

To the best of our knowledge, our study represents the largest cohort of monogenic IEI patients where quantifying MC following a genetic diagnosis by NGS has been assessed. This is also the first study of its kind in Oceania, where advanced healthcare settings and the clinical presentation of IEIs—including infection patterns and local genetics (27, 28, 29)—may differ from that reported in the Northern Hemisphere, from where much of the existing data regarding IEIs is derived. Concomitantly, our findings are likely to be broadly generalizable where in a cohort of more than 200 patients, including 11 identified through NBS, we were able to characterize outcomes across 88 IEIs due to variants in 86 genes (LOF and GOF for *STAT1* and *STAT3*). While a significant percentage of patients identified to have a monogenic diagnosis in our cohort experienced a tangible change in their management and surveillance (84%), the impact of reaching the end of a diagnostic journey in establishing a molecular cause for their disease for all patients in this study cannot be understated. Studies have demonstrated that establishing a genetic diagnosis in the space of rare diseases brings with it a reduction in carer anxiety and psychological burden, improving reproductive confidence, and allows early access to the appropriate support organizations and communities (30, 31, 32, 33). This allows more nuanced prognostication, family planning, and a reduction in the investigative burden experienced, which, when coupled with the 32 family members in our study diagnosed because of the proband, extends these benefits to a much larger number of individuals.

There are some limitations of this study. First, the opt-in model of clinician participation lends to potential self-selection bias that may affect the interpretation and quantification of MC in the cohort. Furthermore, reliance on retrospective clinician recall and case notes may introduce information and recall bias, particularly for timing and nature of MCs. Second, while our analysis spans a period of 12 years, it does not systematically account for trends in NGS availability and costs, NGS technical advancements, NBS practices, or access to therapeutic options, which could confound observed management patterns. Third, our study did not include patients with a clear clinical phenotype strongly suggestive of a specific molecular diagnosis, who would typically be investigated for diagnosis using targeted methods (e.g., candidate gene Sanger sequencing) rather than NGS. This likely accounts for the relative paucity of more common IEIs in our cohort, such as those caused by pathogenic variants in the major X-linked genes such as *IL2RG*, *BTK*, *CD40L*, and *CYBB*, which are routinely identified through targeted molecular sequencing. Finally, we did not perform a financial cost–benefit analysis of NGS outcomes compared to those without a monogenic diagnosis of an IEI, as this was beyond the scope of the current project. However, despite these limitations, the detailed outcomes in >200 patients with 88 IEIs provide a unique, practice informing benchmark for real-world management of IEIs and provide a foundation for future controlled and health economic studies in this group.

In conclusion, the timely identification of monogenic causes of IEIs results in more targeted, pathway-informed management in this clinically heterogeneous cohort. Early availability and subsidized access to NGS, as well as establishing standardized practices, will amplify these benefits on a grater scale. While our data are derived from tertiary hospitals across Oceania, it is important to recognize that many regions in this area remain resource-limited and lack access to NGS. Further studies incorporating cost–benefit analyses of NGS for establishing a definitive diagnosis across diverse healthcare settings in this region are needed to determine the real-world feasibility of this time-critical and increasingly essential diagnostic tool.

## Materials and methods

All CIRCA-affiliated clinicians were invited to participate in a survey collecting de-identified information on patients with monogenic IEIs diagnosed by NGS through any genetic service, via an IEI-focused gene panel, WES, or WGS from January 2013 to December 2025. All variants included were pathogenic; those with autosomal recessive IEIs had two confirmed pathogenic variants in trans, apart from a reported pathogenic dominant negative heterozygous variant in AIRE (34). Indications for genetic testing in these patients included a combination of clinical phenotype, laboratory/immunopathology abnormalities, family history, and/or suspected syndromic associations. Data collected included gender, month and year of birth, clinical presentation, year of IEI diagnosis, management prior to genetic diagnosis, MaMC or MiMC after diagnosis, provision of genetic counseling for family planning, and details of any relatives identified as being affected following establishment of genetic diagnosis in the proband. When family members were diagnosed concurrently, both members were considered probands and were included in our analysis as two individual patients. Patients with diagnoses identified by Sanger sequencing of single genes, as well as cases where variants of unknown significance were identified by NGS, were excluded.

MaMC was defined as targeted or curative therapy being implemented as a direct result of achieving the genetic diagnosis, beyond the standard syndromic recommendations, including identifying/initiating pathway-specific treatment, IRT, recommending and/or guiding HSCT (including conditioning and donor selection), enzyme replacement therapy, and infection prevention with specific prophylactic antimicrobials. MiMC was defined as any other change in management, including increased immune system surveillance, monitoring for organ-specific complications, and other considerations known to be associated with the IEI, such as radiosensitivity. Clinicians were asked to identify whether genetic counseling was sought for family planning following the monogenic diagnosis, and to identify which, if any, family members were diagnosed because of the proband.

Genes were grouped as per the 2024 IUIS classification of IEI: (1) immunodeficiencies affecting cellular and humoral immunity, (2) combined immunodeficiencies with associated or syndromic features, (3) predominantly antibody deficiencies, (4) diseases of immune dysregulation, (5) congenital defects of phagocyte number or function, (6) defects in intrinsic and innate immunity, (7) autoinflammatory disorders, (8) complement deficiencies, (9) bone marrow failure, and (10) phenocopies of IEIs associated with autoantibodies or somatic variants (3). Rate of change of management was calculated by expressing the number of patients with MaMC and/or MiMC as a percentage of all patients diagnosed and included in the study. When no data were provided for MiMC, referral for family planning, or family member diagnoses, the assumption was of no change. 10 patients did not have clinical data and were excluded from the analysis of clinical presentations.

### Online supplemental material

Table S1 summarizes the specific genes in which pathogenic variants were identified, and the number of patients in whom variants were identified for each gene.

## Ethics statement

### Ethics approval

This study was approved in Australia by the Sydney Children’s Hospital Network Human Research Ethics Committee (HREC 2019/ETH12521), and in New Zealand by the Auckland Health Research Ethics Committee approval (AH1284). Patient consent was obtained either at the time of data collection or prior to the genetic testing.

## Supplementary Material

Table S1 shows the number of patients with identified pathogenic variants in specific genes.

## Data Availability

Data are available from the corresponding author upon request.
