# Supplementary material for: Management change following NGS diagnosis of inborn errors of immunity: The Australasian experience
Source: J Hum Immun. 2026 Oct 1;2(6):e20260120. doi: 10.70962/jhi.20260120 (PMC13629484; doi:10.70962/jhi.20260120)
Supplement: Supplementary file 1 [file jhi_20260120_tables1.docx]

***Supplementary table 1:*** ***Number of patients with identified pathogenic variants in specific genes.***

| Gene | No. of patients | Gene | No. of patients | Gene | No. of patients |
| --- | --- | --- | --- | --- | --- |
| *BIRC4* | 8 | *C6* | 2 | *LIG4* | 1 |
| *ADA2* | 6 | *CD40L* | 2 | *MEFV* | 1 |
| *ATM* | 6 | *CHD7* | 2 | *MTHFD1* | 1 |
| *NFKB2* | 6 | *CYBB* | 2 | *MYSM1* | 1 |
| *STAT1* (GOF) | 6 | *FAS* | 2 | *NCF1* | 1 |
| *STING1* | 6 | *FOXP3* | 2 | *NFE2L2* | 1 |
| *WAS* | 6 | *IL2RB* | 2 | *NOD2* | 1 |
| *AIRE* | 5 | *IRAK4* | 2 | *ORAI1* | 1 |
| *CTLA4* | 5 | *MAGT1* | 2 | *OUTLIN* | 1 |
| *DOCK8* | 5 | *PRF1* | 2 | *PLCG2* | 1 |
| *IFNAR1* | 5 | *PTEN* | 2 | *PNP* | 1 |
| *NFKB1* | 5 | *RAG2* | 2 | *POLA1* | 1 |
| *PIK3CD* | 5 | *ACTB* | 1 | *RAC2* | 1 |
| *STAT3* (LOF) | 5 | *ADA* | 1 | *RNU4ATAC* | 1 |
| *TTC7A* | 5 | *ATP6AP2* | 1 | *RORC* | 1 |
| *UNC13D* | 5 | *C4/C5* | 1 | *RUNX1* | 1 |
| *BTK* | 4 | *CARD9* | 1 | *SAMD9L* | 1 |
| *LRBA* | 4 | *CHFR* | 1 | *SBDS* | 1 |
| *RMRP* | 4 | *CSF2RA* | 1 | *SH2D1A* | 1 |
| *STAT3* (GOF) | 4 | *CXCR4* | 1 | *SKIV2L* | 1 |
| *TNFAIP3* | 4 | *DNMT3B* | 1 | *STAT5B* GOF / *KRAS* GOF (s) | 1 |
| *C2* | 3 | *FNIP1* | 1 | *SP110* | 1 |
| *IL2RG* | 3 | *FOXN1* | 1 | *SPINK5* | 1 |
| *KMT2D* | 3 | *HYOU1* | 1 | *STAT1 LOF* | 1 |
| *MVK* | 3 | *IFNGR1* | 1 | *TBX1* | 1 |
| *NLRP3* | 3 | *IL7R* | 1 | *TNFRSF1A* | 1 |
| *PIK3R1* | 3 | *JAK3* | 1 | *TTC37* | 1 |
| *SAMD9* | 3 | *KRAS GOF* (s) | 1 | *ZNF341* | 1 |
| *ARPC1B* | 2 | *LAD1* | 1 |  |  |
| *DCLRE1C* | 2 | *LIG1* | 1 |  |  |

LOF – loss of function, GOF – gain of function, s – somatic variant(s)
